# Open Thoracoabdominal Aortic Procedures following Endovascular Intervention

**DOI:** 10.1055/s-0042-1750117

**Published:** 2022-12-15

**Authors:** Andrea Melloni, Andrea Kahlberg, Enrico Rinaldi, Victor Bilman, Nicola Favia, Germano Melissano, Roberto Chiesa

**Affiliations:** 1 Division of Vascular Surgery, Vita-Salute San Raffaele University, Ospedale San Raffaele, Istituto di Ricovero e Cura a Carattere Scientifico San Raffaele Scientific Institute, Milan, Italy

**Keywords:** aortic aneurysm, endovascular procedure, endoleak, thoracoabdominal

## Abstract

Open conversion of thoracoabdominal aortic (TAA) disease after failed attempts of endovascular treatment is increasingly required. The main causes are endoleak, endograft failure, infection, disease progression, or persistent false lumen perfusion in dissected aortas. Mortality and morbidity rates are high, higher than after standard TAA open repair. Therefore, this surgery should be performed only in dedicated centers by experienced teams. Specific perioperative organ protection protocols, as well as surgical techniques, are crucial to guarantee acceptable results.

## Introduction

Operative treatment of thoracoabdominal aortic (TAA) pathology is increasingly performed by endovascular means. These techniques range from thoracic endovascular aortic repair (TEVAR) with a straight endograft, in some cases associated with visceral revascularization with parallel endografts (so-called chimneys, snorkels, periscopes, and so on), to extensive TAA reconstructions with fenestrated or branched endografts (Fenestrated EndoVascular Aortic Aneurysm Repair, FEVAR, Branched EndoVascular Aortic Aneurysm Repair, BEVAR).

Endovascular strategies aim at reducing operative mortality and morbidity with a less invasive approach. The encouraging results and the continuous advances in technology permit treatment of even older patients with significant cardiac and pulmonary comorbidities, who would be otherwise turned down for an open surgical approach. 1 A recent systematic review and meta-analysis showed similar mortality and postoperative outcomes despite such differences in preoperative characteristics. 2

Long-term durability of TAA endovascular repair remains an open issue. Generally, a significant number of reinterventions are required over time. Most redo procedures are endovascular, from target vessel relining to complete redo FEVAR/BEVAR after failed total endovascular thoracoabdominal repair. 3 Also, endograft durability may be overestimated as age-related and comorbidity-related mortality can hide the impact of endovascular failure. Accordingly, a favorable 91% 5-year freedom from aneurysm-related death must be interpreted in light of the median overall life expectancy of less than 5 years, in a large single-center study. 4

As a consequence of the widespread use of endovascular aneurysm repair (EVAR), the number of patients requiring open conversion (OC) is growing. 5 6 Open surgery after failed endovascular repair is more challenging than in native aortas, with the technical issues of endovascular material removal and management of fragile vascular tissues. These factors contribute to the higher in-hospital mortality in these patients. 7 As only few studies have previously reported OC after TAA endovascular repair, the aim of this paper is to describe specific features and different scenarios, as well as to provide technical surgical tips emanating from our single-center experience with OC after TEVAR, FEVAR, and BEVAR.

## Indication for Open Conversion

**Fig. 1:**
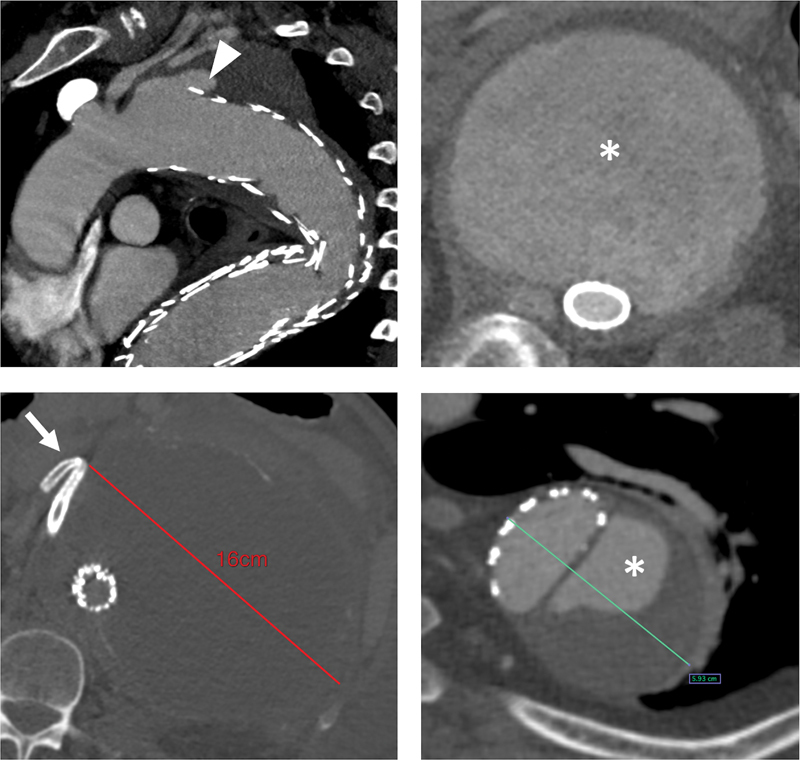
Common causes of open conversion after endovascular thoracoabdominal repair. *Top left* : Type Ia endoleak ( *arrowhead* ) after extent II fenestrated/branched endovascular aortic aneurysm repair (BEVAR); *bottom left* : giant thoracoabdominal aortic aneurysm with persistent growth after BEVAR and dislodgement of the bridging stent for the right renal artery ( *arrow* ); *right* : persistent false lumen (*) perfusion and aneurysm enlargement after endovascular repair with multilayer flow modulator devices ( *top* ) or thoracic endovascular aortic repair ( *bottom* ).

Late OC after prior endovascular repair may be required to solve several conditions affecting the treated aorta, the adjacent aortic segments, the endovascular devices themselves, or a combination of these factors. Specific classifications have previously been proposed to group indications for OC (repair failure or extension 8 and disease- or endograft-related failure 6 ). The 2021 Society for Vascular Surgery Reporting Standards for endovascular TAA aneurysm repair differentiate between aortic disease progression, device failure (migration >10 mm, device degradation, loss of device integrity), or endoleak (EL). 9 Possible long-term complications requiring OC ( Fig. 1 ) include:

- EL with proximal EL (Type Ia) being the most common indication for OC after TEVAR, 10 and Type II EL a common cause of conversion after EVAR.
- Endograft fracture/migration.
- Infection, accounting for approximately 10% of OC cases in reported series. 5 6 7
- Proximal/distal disease progression, with distal progression being the leading cause of conversion in series with a high proportion of postdissecting aortic disease. 7
- Persistent false lumen perfusion, limited to postdissection patients, not infrequently needing late OC after the initial endovascular treatment, 7 11 especially if the original treatment was a simple TEVAR.

## Patients' Characteristics

The need for TAA open repair after previous attempts at endovascular treatment may affect people of all ages, with series reporting patients treated from their second to their ninth decade of life. 6 7 12 Given the increased technical complexity and higher postoperative mortality after OC compared with native aortic surgery 7 and the issue of long-term durability, physicians must carefully evaluate the relative benefits of endovascular procedures in young fit patients. They must consider a sustained aortic-related survival, not simply achieving a low 30-day complication rate, which must be considered as the main treatment aim in this cohort. Most patients are men, of whom a variable portion (2–49%) is affected by connective tissue disease (CTD), which is associated with a higher risk of endovascular failure 13 and has traditionally been considered a contraindication to endovascular treatment, favoring primary open repair.

Many patients eventually undergo OC after multiple surgical or endovascular procedures. Frequently, OC represents the last hope for these patients. Repeated contrast administration for diagnostic or therapeutic procedures is associated with the risk of acute renal injury and chronic kidney disease, which is notably associated with worse downstream outcomes after TAA surgery. 14 Moreover, previous sternotomy or thoracotomy for both coronary artery disease or ascending/aortic arch surgery may increase cardiopulmonary morbidity after open TAA surgery and must be considered in the decision-making process.

Finally, endograft infection is per se a condition with a very poor prognosis. Frequently, these patients undergo OC either urgently or emergently (e.g., for hemorrhagic shock due to an aorto-esophageal fistula) or weakened after a long period of hospitalization and antibiotics. Optimization of nutritional status and exercise capacity is rarely feasible. When outcomes are analyzed separately depending on the indication for admission, this cohort is burdened by the highest mortality, even in top-ranking centers. 5 7

## Technical Considerations

### Patient Preparation and Surgical Exposure

Open repair of TAA disease is burdened with considerable mortality and permanent paraplegia rates, reaching respectively up to 8.9 and 4.4%, but the latter can be as high as 15% in extent II aneurysms. 2 15 Organ protection at our institution is multimodal and has previously been described in detail in dedicated publications. This includes distal perfusion strategies, often by means of left heart bypass, selective renovisceral vessel perfusion, 16 optimal management of blood products, 17 and cerebrospinal fluid drainage with an automated pressure-controlled device (in all extent I–II–III thoracoabdominal aortic aneurysms [TAAAs] and selectively in extent IV). 18 Spinal cord injury (SCI) prevention protocols should be carefully tailored to every single patient, bearing in mind that multiple previous endovascular interventions may increase the risk of SCI (as the consequence of an impaired collateral network) although staging may at times serve as a form of spinal cord preconditioning. 19

Open TAA repair is best performed through a left thoraco-phreno-laparotomy to expose the entire thoracic and abdominal aorta and obtain access to safe proximal and distal clamping sites. In comparison to naïve aortas, the presence of endovascular material may require a wider exposure of the aorta to clamp an endograft-free segment when complete removal is deemed necessary. The entry intercostal space is usually the fifth or sixth but varies from the fourth to the eighth according to disease extent. Occasionally, a bilateral subcostal incision can be performed instead, when supraceliac clamping is sufficient to perform the repair. Aortic exposure in patients with chronically dissected aortas or previous endovascular repair for symptomatic/ruptured aneurysms typically requires prolonged and gentle lysis of pleural adhesions. This is particularly true after a previous sternotomy (e.g., after ascending/aortic arch repair). Avoiding direct lung damage and performing a limited phrenotomy are crucial factors to prevent postoperative pulmonary complications, which are not uncommon in this subset of patients.

As far as the paravisceral aortic portion is concerned, careful exposure of the first few centimeters of the renovisceral vessels may help to selectively cross-clamp them before insertion of perfusion cannulas and to perform additional maneuvers like endarterectomy or stenting. As a matter of fact, previous endovascular aortic repair at this segment may include bridging stents extending far from the artery orifice and stent removal, endarterectomy, or other adjunctive procedures may be needed.

### Endograft Removal

**Fig. 2:**
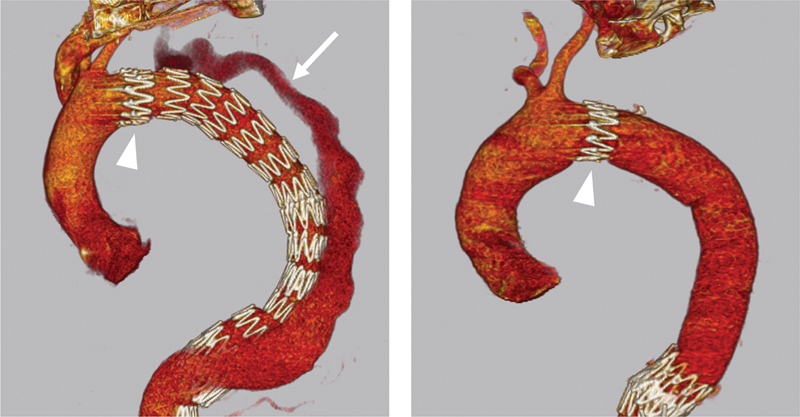
*Left* : persistent false lumen perfusion ( *arrow* ) after TEVAR and true lumen re-expansion with the PETTICOAT technique. *Right* : open conversion with partial removal of the thoracic endograft. A surgical graft was anastomosed proximally to the first stent of the endograft ( *arrowhead* ) and distally to the medio-thoracic aorta above the bare-metal stents. PETTICOAT, provisional extension to induce complete attachment; TEVAR, thoracic endovascular aortic repair.

The most obvious difference between primary open TAA surgery and OC is the presence of endovascular material that must be removed before proceeding to the aortic reconstruction. Previously deployed aortic endografts may be removed either partially or totally, according to the initial indication for OC and to anatomical/technical considerations. Stent-graft infection is typically accepted as an indication for complete endograft removal to eradicate the septic focus. 20 Total endograft removal may be considered also in patients with CTD in whom endovascular treatment was performed as a bridging procedure in a life-threatening situation. 10 In case the previous endograft extends proximally within the aortic arch, so that clamping proximal to the endoprosthesis is impossible, hypothermic circulatory arrest can be considered. Well-incorporated endovascular material can be partially left in place in the absence of local infection and if adequate aneurysm exclusion and sealing is provided in that specific aortic segment. This is typically the case for an infrarenal EVAR with adequate sealing in the iliac zones but proximal EL (or vice-versa a proximal TEVAR with distal sealing issues). In such cases, the endograft can be trimmed with a scalpel and stent struts can be cut at the level of the intended graft-to-endograft anastomosis ( Fig. 2 ). 6 Total endograft preservation can occasionally be performed when the progression of disease is the reason for surgery rather than device failure itself (e.g., development of extent III TAAA after TEVAR for a descending thoracic aortic aneurysm).

**Fig. 3:**
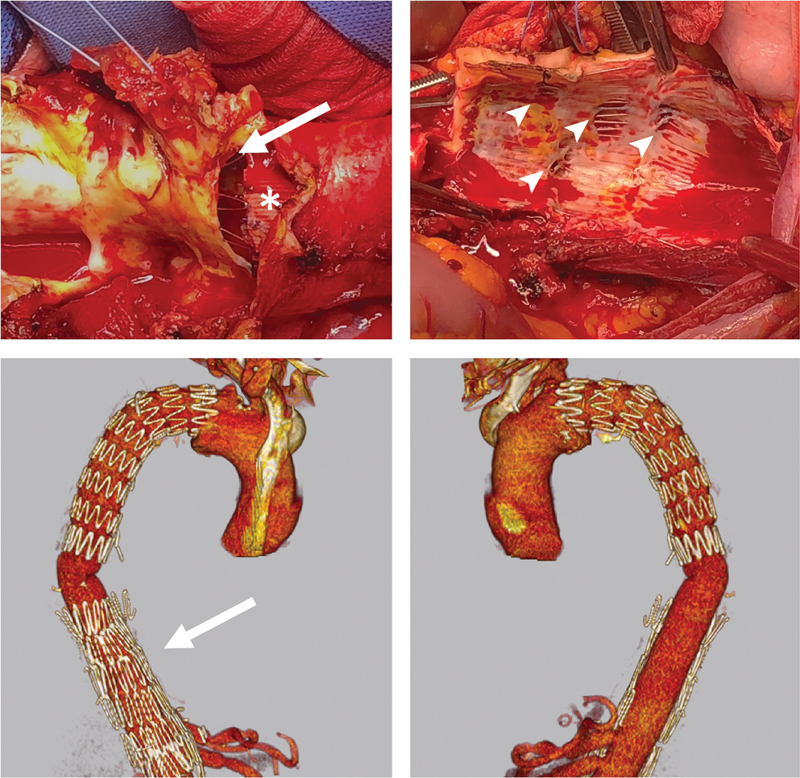
*Top* : Open conversion after PETTICOAT with preservation of the thoracic endograft (*) and partial removal of the bare-metal stents ( *arrow* ) at the level of the renovisceral arteries ( *arrowheads* ). *Bottom* : postoperative computed tomography angiography with the stents left in place on the right aspect of the aorta ( *arrow* ) and completely removed on the left side. PETTICOAT, provisional extension to induce complete attachment.

**Fig. 4:**
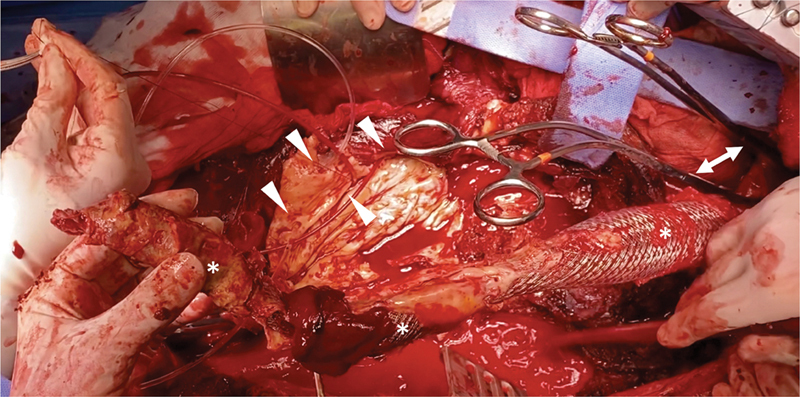
Emergent open conversion for a ruptured extent II thoracoabdominal aortic aneurysm previously treated with multilayer flow-modulator (MFM) devices: the MFM (*) is provisionally clamped with two reinforced clamps ( *arrows* ). The MFM is removed to expose the renovisceral vessels for cannulation ( *arrowhead* ).

A less obvious but technically demanding scenario is OC due to persisting false lumen perfusion and aneurysm enlargement after bare-stent positioning for TAA dissection (e.g., PETTICOAT technique—provisional extension to induce complete attachment) or multilayer flow-modulator (MFM) devices. 21 22 In the first case, late OC can become necessary after several years from the index procedure. The stent struts frequently become tightly encased in the aortic wall and complete stent removal is rarely feasible without performing simultaneous aortic endarterectomy, entailing the risk of compromising the ostia of visceral vessels, or damaging the surrounding structures. In such cases, approached from a left thoraco-phreno-laparotomy, a partial removal can be conducted, leaving the stent in place in the medial aspect of the aorta to avoid esophageal damage ( Fig. 3 ). The renovisceral vessels are revascularized in most cases using the dedicated branches of a multibranched presewn Dacron graft. In the presence of MFM devices, the closed-cells design implies two consequences: the rigid stent structure prevents efficient clamping of the device, so proximal aortic cross-clamping must be sought in an MFM-free aortic segment; moreover, a certain degree of thrombosis of renovisceral arterial ostia is often present and careful vessel preparation after MFM removal must be performed before surgical reconstruction ( Fig. 4 ).

### Thoracoabdominal Aortic Reconstruction

#### Proximal

**Fig. 5:**
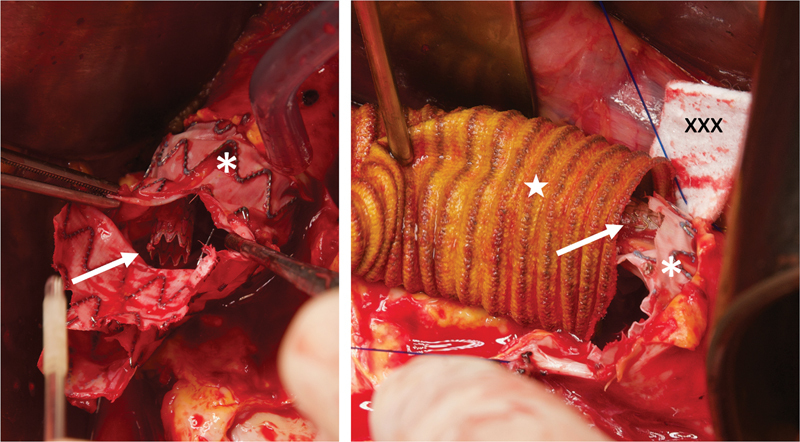
Semiconversion after fenestrated endografts for a pararenal aortic aneurysm. *Left* : partial stentgraft (*) removal and trimming. The bridging stent for the right renal artery ( *arrow* ) is left in place. *Right* : proximal anastomosis of an aorto-bifemoral surgical graft. The triple-layer suture including the previous endograft (*), the surgical graft (★) and a Teflon felt (xxx) to create a neo-neck, with the bridging stent ( *arrowhead* ) for the right renal artery included in the reconstruction.

According to the partial or full removal of endovascular material, the proximal end-to-end anastomosis is usually performed in a triple or quadruple layer with a 2/0 or 3/0 polypropylene running suture, with the inclusion of the previous endograft (if present), the new surgical graft, the aortic tissue, and an outer Teflon felt to create a neo-neck ( Fig. 5 ). If the distal stent of the endograft must be included in the suture, great care must be paid to place the struts inside the Dacron graft, to avoid oozing from the anastomosis, which is difficult to correct after unclamping.

#### Distal

Distal anastomosis during TAA reconstruction is usually performed at the level of the aortic bifurcation with an end-to-end anastomosis using a 2/0 or 3/0 polypropylene running suture reinforced with Teflon pledgets or felt. More distal reconstructions during the same procedure are possible, but more challenging to perform with the thoraco-phreno-laparotomic access needed for visceral reconstruction. In native anatomies, in the presence of bilateral iliac aneurysms, we prefer to perform the distal reconstruction in a second procedure through a midline laparotomy, to guarantee better access to the hypogastric arteries (that are carefully preserved to maintain adequate spinal cord perfusion). If the iliac arteries are healthy and adequate sealing is provided in the presence of an infrarenal EVAR (standard or with mono- or bilateral iliac branch devices), a neo-carrefour can be created to serve as the site of distal anastomosis. After endograft partial removal, the iliac limb endografts are trimmed at the level of the iliac arteries ostia and a 4/0 running suture is performed between the iliac endograft limbs, simultaneously securing them to the native iliac arteries. Endoclamping with Foley or Pruitt catheters prevents fabric and stent disruption associated with external cross-clamping and could be useful in this setting.

### Visceral Vessels

To better deal with visceral vessels, which frequently need some degree of preparation in OC (thrombectomy, endarterectomy, stent/stentgraft removal or trimming, etc.), and to avoid reoperation due to late degeneration of a visceral patch, we prefer to revascularize the renovisceral vessels with end-to-end single bypasses. Usually, a presewn multibranched surgical prosthesis is employed to speed up the reconstruction, but additional grafting can be necessary in case of multiple renal arteries or other anatomic variants. 23 If OC is performed after BEVAR, bridging stents are partially removed to expose the artery ostium. The proximal portion of the remaining bridging stent can be sutured with two to four 6/0 sutures to fix it to the artery. The distal end-to-end anastomosis is then performed at this level.

### Intercostal Arteries

The decision to reattach intercostal arteries to prevent spinal cord ischemia during TAAA repair is still a controversial matter of debate. Preoperative surgical planning should include computed tomography angiography evaluation of patency and diameter of intercostal arteries for each vertebral segment, as well as identification of the *arteria radicularis magna* (artery of Adamkiewicz). In patients needing OC after previous endovascular repair, some peculiarities may influence the decision to proceed with the reimplantation or not. On the one hand, prior endovascular repair could work as a preconditioning factor: several segmental arteries are excluded from the circulation during the index procedures, whether by a TEVAR or due to false lumen thrombosis or by initial aneurysm exclusion in cases of complete TAAA treatment by F/BEVAR. In this setting, intercostal artery reattachment could be regarded as superfluous, as the increased complexity of the reconstruction would probably bring little benefit to the patient. On the other hand, in this notably high-risk surgery, reimplantation of spinal cord feeders could increase resilience to ischemia occasioned by postoperative events such as a sudden hypotension. Motor evoked potentials and somato-sensory evoked potential (MEPs/SSEP) intraoperative monitoring can guide the surgical team in deciding whether or not to reattach the intercostal arteries. Concomitant anesthesia maneuvers such as increasing arterial pressure and increasing cerebrospinal fluid drainage are often taken concurrently. An aggressive policy of critical (T8–T12) segmental artery reattachment is, therefore, recommended, when technically feasible based on preoperative imaging and intraoperative findings (SSEP/MEP deficits or poor backflow [which can indicate poor collateralization]) to avoid this disabling complication, especially in young patients. 24

#### Associated Nonvascular Surgical Procedures

TAA reconstruction with renovisceral and intercostal artery reattachment is obviously the core of OC after failed endovascular repair. However, given the complexity of this surgery, associated nonvascular procedures may be required. Notably, in the largest open TAA series published thus far, up to 12% of patients underwent splenectomy and 2% cholecystectomy during TAAA repair. 14

OC requires dedicated organ reconstruction when it is performed for endograft infection. In the presence of aorto-esophageal, aorto-duodenal, or aorto-bronchial fistula, simple vascular reconstruction without adequate fistula management is associated with poor prognosis. 25 Complementary procedures may be performed together with the aortic reconstruction or in a second stage and include:

- Esophageal direct repair or esophageal resection/stripping, closure of the cervical esophagus, and preparation of a nutritional gastro- or jejunostomy, followed by delayed gastrointestinal reconstruction.
- Duodenal direct repair or resection and anastomosis.
- Bronchial direct repair or resection and anastomosis or wedge pulmonary resection.

Any vascular graft and vascular anastomosis placed in an infected field should always be covered by viable tissue according to the specific setting, such as intercostal muscle flap or pericardium in the thoracic aorta or pedicled omentum in the abdomen. In the absence of such structures, a bovine pericardial patch can be used to prevent contact between the aortic graft and nearby organs. Given the complexity and variety of such reconstructive surgery, dedicated multispecialty teams should be created in tertiary centers that can work as regional or national referral hubs. Multicenter international registries should help increasing knowledge regarding the best therapeutic options for this complex cohort of patients.

## San Raffaele Hospital Experience

Our institution is a tertiary teaching hospital and a referral center for thoracoabdominal open aortic surgery, not infrequently treating patients who need OC after failed TAA endovascular repair. The 15-year experience with OC of thoracic and abdominal aortic endovascular treatment has recently been summarized in a publication describing the intraoperative and 30-day results. 6 The updated cohort is composed of 136 patients who received OC after abdominal EVAR and 88 after EVAR of the thoracic aorta or thoracoabdominal EVAR.

The most common indication for OC was EL in both groups, although graft infection impacted up to 21% of thoracic OC and 10% of the abdominal OC, higher than other contemporary reports. 7 Thirty-day mortality ranged from 0 to 33%, according to indication for OC, the highest mortality being for retrograde aortic dissection and thoracic endograft infection.

## Conclusion

OC after failed endovascular treatment of TAA disease is increasingly performed, often with high perioperative mortality and morbidity rates. Besides patient selection and optimization, a meticulous surgical technique regarding endograft removal and aortic and visceral arterial reconstruction in experienced tertiary centers is crucial to guarantee acceptable results.
